# Endemic potential of Chagas disease in the U.S. southwest: updated surveillance of infected Triatomines from the U.S.-Mexico border region

**DOI:** 10.1017/S095026882510071X

**Published:** 2025-10-27

**Authors:** Priscila Silva Grijo Farani, Austin Fritz, Amanda Faier Pereira, Marina da Silva Ferreira, Sayonara de Melo Viana, Celinda Crews, Rosa A. Maldonado

**Affiliations:** 1 Department of Biological Sciences, The University of Texas at El Paso College of Science, El Paso, TX, USA; 2 Department of Pharmaceutical Sciences, School of Pharmacy, The University of Texas at El Paso College of Science, El Paso, TX, USA; 3 Platform of Molecular analysis, Laboratory of Molecular Virology and Parasitology, Oswaldo Cruz Institute, Oswaldo Cruz Foundation, Rio de Janeiro, Brazil

**Keywords:** Chagas disease, El Paso, Texas, Triatomines, *Trypanosoma cruzi*

## Abstract

*Trypanosoma cruzi*, the etiological agent of Chagas disease, is a vector-borne parasite traditionally associated with sylvatic environments. We investigated the prevalence of *T. cruzi* in triatomines collected from El Paso County, Texas, and southern New Mexico. Specimens were morphologically identified as *Triatoma rubida* and subjected to quantitative PCR for parasite detection. Molecular sequencing of satellite and microsatellite DNA targets was performed to confirm species identity and assess strain lineage. Infected vectors were collected from both sylvatic and urban locations, including Franklin Mountains State Park and residential areas in El Paso (TX) and Las Cruces (NM). Of the 26 triatomines tested, 88.5% were positive for *T. cruzi*, representing a significant increase compared to a previous regional study, which reported an infection rate of 63.3%. The high prevalence of *T. cruzi*-infected *T. rubida*, particularly in urban and peri-urban areas of El Paso and Las Cruces, underscores the increasing public health significance of Chagas disease along the U.S.–Mexico border. These findings highlight the urgent need for sustained vector surveillance, advanced molecular characterization, and focused public health interventions to reduce transmission risks and raise clinical awareness in affected regions.

## Introduction

Chagas disease, formally known as American trypanosomiasis [1], is a potentially life-threatening parasitic disease caused by the protozoan parasite *Trypanosoma cruzi.* It is primarily transmitted to humans and other mammals by triatomine insects, commonly referred to as ‘kissing bugs’. Although historically endemic to Latin America, Chagas disease has become a growing public health concern in non-endemic regions due to population mobility and emerging autochthonous transmission [2, 3]. The World Health Organization estimates that approximately 6–7 million people are infected globally, with increasing cases reported outside endemic countries [4]. In the United States, it is estimated that over 300,000 people are living with *T. cruzi* infection, the majority of whom are immigrants from endemic areas [5, 6]. However, these figures are likely underestimated due to the non-mandatory reporting of Chagas disease in most states, limited access to diagnostic testing, and a lack of awareness among healthcare providers and the general public [7, 8].

Over the past two decades, research has increasingly documented the presence of *T. cruzi*-infected triatomine vectors across the southern United States, including Florida, Louisiana, Texas, Arizona, New Mexico, and California [9–12]. These studies have identified both sylvatic and peridomestic transmission cycles involving wildlife, domestic animals, and humans, raising concerns about the potential for autochthonous transmission [13]. Infected vectors have been collected in diverse ecological settings, ranging from rural to urban environments, highlighting the adaptability of the parasite and its vectors to changing landscapes. The expanding body of evidence underscores the need for continued vector surveillance and ecological studies to assess the risk of human infection and inform public health strategies [13–15].

In the United States, approximately ten triatomine species have been recorded [16], several of which have been implicated in *T. cruzi* transmission. However, southern U.S. states such as Texas, New Mexico, and Arizona share borders with Mexico, a country with significantly greater triatomine diversity and broad vector distribution [17]. The proximity to regions with elevated entomological risk, combined with shared ecological corridors and human mobility, increases the vulnerability of U.S. border states to vector introduction, hybridization, and potential changes in vector behaviour and transmission dynamics [16, 18]. Additionally, recent taxonomic revisions within the *Triatoma protracta* species complex, such as the recognition of Hospesneotomae as a new genus, highlight the evolving nature of triatomine systematics and the importance of sustained taxonomic and entomological surveillance [19]. These findings reinforce the need for sustainable and regionally tailored vector monitoring strategies across the U.S.–Mexico border region.

Moreover, over the past decade, research efforts have increasingly focused on detecting *T. cruzi* in the El Paso, Texas, border region. A comprehensive surveillance study involving stray dogs and cats, sylvatic mammals, and triatomine insects confirmed the existence of a sylvatic transmission cycle of *T. cruzi* in the El Paso area [20]. As human encroachment into wildlife habitats intensifies and urbanization expands, evaluating the infectivity of vectors has become critical for understanding and monitoring the local transmission potential of Chagas disease. In this context, nearly seven years after our group’s initial surveillance study of wild animals and triatomine vectors, we present an updated assessment of triatomines collected in the region and tested for *T. cruzi* infection.

## Materials and methods

### Ethics statement and collection sites

Triatomines were collected from wild and peridomestic environments within urban areas of the El Paso region, Texas, over a 10-month period between April 2024 and March 2025. Collections were conducted every two weeks using CDC light traps set for 24-hour periods, with sampling frequency adjusted to field conditions and accessibility [21, 22]. Beginning 19 May 2024, triatomines were detected within the capture sleeves of the traps. The traps were deployed approximately 1 metre above the ground in a rugged, arid habitat characterized by multiple arroyos and an average elevation of 1500 metres. Vegetation at the sites included native desert flora such as cacti, yuccas, sotol, ocotillo, and creosote bush. At one trap site, adult triatomines were also directly collected from the adobe-like walls of a bird blind, a human-constructed shelter that mimics natural structures. The overnight release of carbon dioxide from the CDC light traps may have contributed to the increased presence of specimens near the bird blind. Animal life in the region includes small mammals, coyotes, foxes, mule deer, mountain lions, and a wide variety of bird and reptile species. No nymphal stages were collected during this sampling effort. Sample collection and processing adhered to ethical guidelines and were conducted under protocol number 1608423-2, approved on May 5, 2023, by the Institutional Biosafety Committee (IBC).

### Identification of triatomine species

Triatomine specimens were identified to the species level using morphological characteristics examined under a stereoscope. Identification was performed with the aid of a dichotomous key adapted by Bejcek [23], which is based on the foundational taxonomic revision of Triatominae by Lent and Wygodzinsky [24] [23, 24]. Diagnostic features used for species determination included head shape, pronotum structure, leg morphology, connexivum coloration, and other species-specific traits.

The dichotomous key by Bejcek was specifically developed to differentiate North American Triatominae species, including *T. rubida*, from morphologically similar species and potential misidentifications. Reference illustrations and comparative morphological criteria described in the original revision by Lent and Wygodzinsky were used to support identification accuracy.

### Quantitative multiplex real-time PCR (qPCR) assays

Digestive tracts were dissected from collected *T. rubida* specimens and incubated for 2 hours at 56 °C in 200 μL of lysis buffer containing 10 mM Tris-HCl (pH 9.2), 1 mM EDTA, and 150 μg/mL proteinase K (Roche Diagnostics GmbH, Mannheim, Germany). Genomic DNA was extracted using the High Pure PCR Template Preparation Kit (Roche Diagnostics GmbH, Mannheim, Germany) and eluted in 100 μL of the elution buffer provided in the kit, as previously described [25]. DNA was stored at – 20°C until further analysis. Multiplex quantitative real-time PCR (qPCR) was performed in a final reaction volume of 20 μL, containing 2 μL of extracted DNA (8–10 ng), 1× FastStart TaqMan Probe Master Mix (Roche Diagnostics GmbH, Mannheim, Germany), 600 nM Cruzi1 and Cruzi2 primers, and 250 nM Cruzi3 probe (FAM/NFQ-MGB) targeting *T. cruzi* nuclear satellite DNA (SatDNA). For internal amplification control, the reaction also included 300 nM P2B primer, 500 nM P6R primer, and 150 nM Triat probe (VIC/NFQ-MGB; Applied Biosystems, Foster City, CA, USA) targeting the 12S ribosomal RNA gene of triatomines, as previously described [25, 26]. Thermal cycling was performed on a QuantStudio 3 Real-Time PCR System (Applied Biosystems, Foster City, CA, USA) with the following cycling conditions: 50 °C for 2 min, 95 °C for 10 min, followed by 40 cycles of 95 °C for 15 s and 58 °C for 1 min.

### Molecular identification by DNA sequencing

Samples yielding positive PCR amplicons for *T. cruzi* SatDNA were excised from agarose gels and purified using the Wizard® SV Gel and PCR Clean-Up System (Promega), following the manufacturer’s instructions. Purified amplicons were sequenced at Plasmidsaurus Inc. DNA sequences were analysed using NCBI BLAST, confirming sequence identity based on query alignment criteria [20].

### Geographic mapping of triatomine collection sites

The locations of triatomines collected were documented, and the coordinates of collection sites were provided. The software Land Lease Viewer (https://gisweb.glo.texas.gov/glomapjs/index.html) was used to generate a geospatial map of collection sites.

## Results

### Prevalence of T. cruzi in collected triatomines

**Figure 1.**
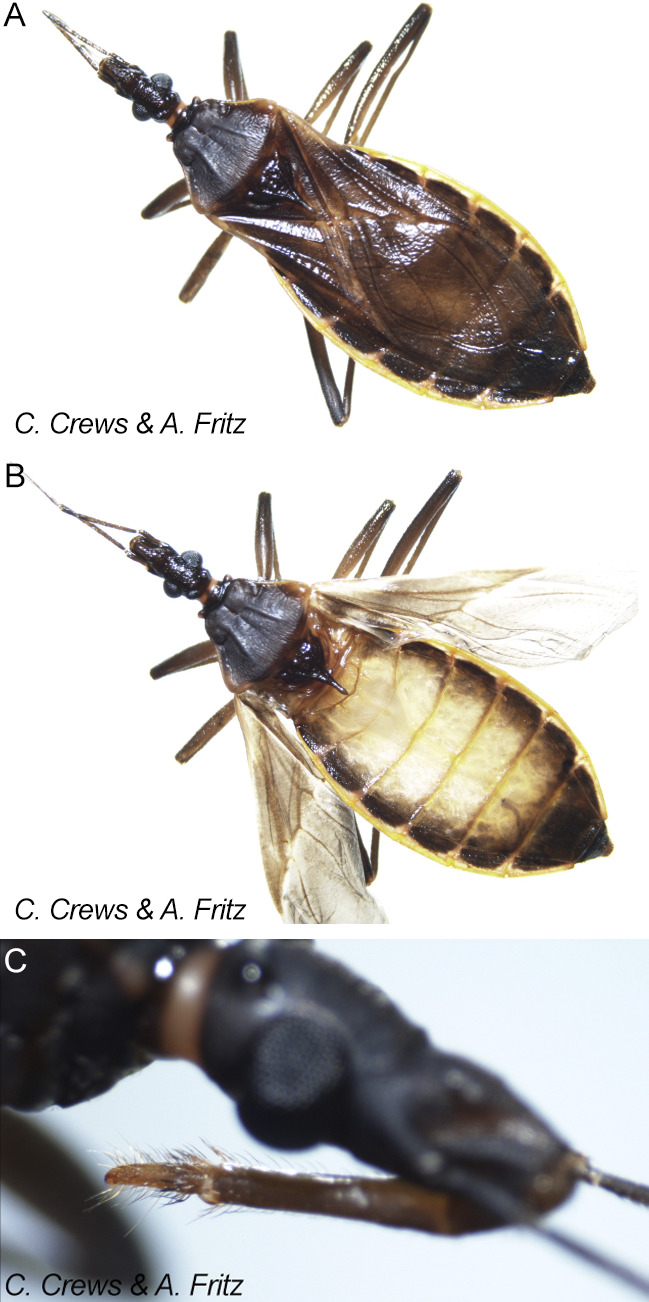
Adult *Triatoma rubida* specimens collected in the EL Paso region, Texas. (A) Dorsal view of an adult *T. rubida*, highlighting the dark brown to black body coloration and the characteristic orange-yellow marginal markings along the connexivum. (B) Dorsal view with partially extended wings, revealing the membranous hindwings and striped abdominal tergites. (C) Close-up of the head and thorax, showing the compound eye, antennal segments, and base of the rostrum. All specimens were collected using CDC light traps set at approximately 1 metre above ground level. Photos by C. Crews & A. Fritz.

All triatomine specimens were collected from either El Paso County, Texas, or southern New Mexico. In Franklin Mountains State Park, Texas, triatomines were collected from sylvatic habitats including hiking trail perimeters, dry arroyos, exposed rock piles, crevices between boulders, and beneath desert shrubs such as lechuguilla (*Agave lechuguilla*), creosote bush (*Larrea tridentata*), and sotol (*Dasylirion spp.*). In urban El Paso (sites EP-S1 and EP-S2), specimens were found near residential building perimeters, often under shaded debris or outdoor wooden structures such as stored firewood and garden furniture. In Las Cruces, New Mexico (LC-S1), one specimen was collected in a semi-rural environment, adjacent to an open garage structure and discarded wooden pallets. These habitats represent typical triatomine refugia, particularly for adult *T. rubida*, which are known to seek shelter in shaded crevices and emerge during nocturnal hours to seek hosts. All specimens were morphologically identified as *T. rubida* (Figure 1) and categorized by life stage, sex, and geographic origin. Collection sites included both sylvatic habitats within Franklin Mountains State Park and urban or peri-urban environments outside the park, specifically Las Cruces Site 1 (LC-S1) and El Paso Sites 1 and 2 (EP-S1, EP-S2).

**Table 1.** Prevalence of *T. cruzi* in triatomines collected in El Paso County and New Mexico

Sample ID	Species	Life stage	Gender	Location name	Location coordinates	*T. cruzi*
1	*T. rubida*	Adult	Female	Franklin Mountains Bird Blind (FM-BB)	31.922; −106.514	Negative
2	*T. rubida*	Adult	Female	Franklin Mountains Bird Blind (FM-BB)	31.922; −106.514	Positive
3	*T. rubida*	Adult	Female	Franklin Mountains Bird Blind (FM-BB)	31.922; −106.514	Positive
4	*T. rubida*	Adult	Male	Franklin Mountains Bird Blind (FM-BB)	31.922; −106.514	Negative
5	*T. rubida*	Adult	Male	Franklin Mountains Bird Blind (FM-BB)	31.922; −106.514	Positive
6	*T. rubida*	Adult	Female	Franklin Mountains Interpretive Center (FM-IC)	31.910; −106.518	Positive
7	*T. rubida*	Adult	Female	Franklin Mountains Interpretive Center (FM-IC)	31.910; −106.518	Positive
8	*T. rubida*	Adult	Male	Franklin Mountains Interpretive Center (FM-IC)	31.910; −106.518	Positive
9	*T. rubida*	Adult	Female	Franklin Mountains Visitor Center (FM-VC)	31.910; −106.518	Negative
10	*T. rubida*	Adult	Female	Franklin Mountains Visitor Center (FM-VC)	31.910; −106.518	Positive
11	*T. rubida*	Adult	Female	Franklin Mountains 1 (FM-T1)	31.910; −106.518	Positive
12	*T. rubida*	Adult	Female	Franklin Mountains 1 (FM-T1)	31.910; −106.518	Positive
13	*T. rubida*	Adult	Male	Franklin Mountains 2 (FM-T2)	31.913; −106.516	Positive
14	*T. rubida*	Adult	Female	Franklin Mountains 2 (FM-T2)	31.913; −106.516	Positive
15	*T. rubida*	Adult	Female	Franklin Mountains 2 (FM-T2)	31.913; −106.516	Positive
16	*T. rubida*	Adult	Female	Franklin Mountains 4 (FM-T4)	31.910; −106.513	Positive
17	*T. rubida*	Adult	Female	Franklin Mountains 4 (FM-T4)	31.910; −106.513	Positive
18	*T. rubida*	Adult	Male	Franklin Mountains 4 (FM-T4)	31.910; −106.513	Positive
19	*T. rubida*	Adult	Male	Franklin Mountains 5 (FM-T5)	31.926; −106.513	Positive
20	*T. rubida*	Adult	Female	Franklin Mountains 5 (FM-T5)	31.926; −106.513	Positive
21	*T. rubida*	Adult	Female	Franklin Mountains 5 (FM-T5)	31.926; −106.513	Positive
22	*T. rubida*	Adult	Male	Franklin Mountains 5 (FM-T5)	31.926; −106.513	Positive
23	*T. rubida*	Adult	Female	Franklin Mountains 5 (FM-T5)	31.926; −106.513	Positive
24	*T. rubida*	Adult	Female	Las Cruces Site 1 (LC-S1)	32.315; −106.858	Positive
25	*T. rubida*	Adult	Female	El Paso Site 1 (EP-S1)	31.774; −106.503	Negative
26	*T. rubida*	Adult	Male	El Paso Site 2 (EP-S2)	31.774; −106.484	Positive

Of the 26 triatomines tested, 22 (84.6%) were positive for *T. cruzi* DNA by quantitative PCR. Infected vectors were identified at nearly all sampling locations, with the highest number of positive specimens originating from sylvatic sites such as the Franklin Mountains Bird Blind (FM-BB), Interpretive Center (FM-IC), and transect sites FM-T2 and FM-T5. Both male and female specimens were infected, indicating no apparent sex-based difference in prevalence. Importantly, *T. cruzi*-positive triatomines were also detected at El Paso urban collection sites (EP-S1 and EP-S2), highlighting the encroachment of infected vectors into areas with direct human exposure. This migration into urban zones represents a significant epidemiological concern, as it expands the potential for transmission from traditionally sylvatic cycles to domestic settings. Compared to a previous study conducted in the region, which reported a 63.3% infection rate [20], the current prevalence of 84.6% represents a marked increase. This rise may reflect improvements in detection sensitivity, changing environmental conditions, or an actual intensification of *T. cruzi* transmission.

### DNA sequencing analysis

Sequencing analysis was performed on PCR-positive *T. cruzi* samples to confirm parasite identity and explore potential genetic diversity. All sequences aligned with *T. cruzi* satellite or microsatellite DNA targets, with identity values ranging from 96.95% to 100% and E-values from 3.00E–69 to 3.00E–78, confirming the specificity of the molecular assay.

The sequences matched various known *T. cruzi* clones, including R10I, R20D, R2G, R4J, R8I, and R9S, as well as microsatellite loci such as cl13.1, cl16.1, and cl20.1 (e.g., clone Las Palomas 163). These reference sequences are typically associated with the TcI lineage, the most widely distributed discrete typing unit (DTU) in North and Central America (Table 2; Supplementary Table 1). However, discrete typing unit (DTU) genotyping could not be performed due to the low parasite burden in the samples, which impeded the successful amplification of multilocus genotyping targets, as outlined by Moreira & Ramirez [27].

**Table 2.** Molecular identification of *T. cruzi* in *T. rubida* triatomines collected in El Paso County and New Mexico, based on sequence alignment (E-value and percent identity)

Sample ID	Accession number	Description	E-Value	Identity %
2	OM730064.1	*T. cruzi* clone R20D satellite sequence	6.00E−76	99.39
3	AY520032.1	*T. cruzi* clone ClBF06 satellite sequence	3.00E−69	96.95
5	FJ768483.1	*T. cruzi* strain 115 clone B09 satellite sequence	6.00E−76	99.39
6	AY520019.1	*T. cruzi* clone ClC18c12 satellite sequence	1.00E−77	100.00
7	AY520077.1	*T. cruzi* clone YE01 satellite sequence	3.00E−74	98.78
8	AY520038.1	*T. cruzi* clone ClBD07 satellite sequence	6.00E−71	97.56
10	OM730061.1	*T. cruzi* clone R10I satellite sequence	3.00E−78	100.00
11	OM730064.1	*T. cruzi* clone R20D satellite sequence	6.00E−71	97.56
12	OM730049.1	*T. cruzi* clone R2H satellite sequence	1.00E−77	100.00
13	OM730061.1	*T. cruzi* clone R10I satellite sequence	3.00E−78	100.00
14	LT220316.1	*T. cruzi* microsatellite DNA locus cl13.1, clone cl13.1	1.00E−77	100.00
15	LT220332.1	*T. cruzi* microsatellite DNA locus cl16.1, clone Las Palomas 163	3.00E−69	96.95
16	OM730048.1	*T. cruzi* clone R2G satellite sequence	1.00E−77	100.00
17	OM730060.1	*T. cruzi* clone R9S satellite sequence	3.00E−74	98.78
18	OM730052.1	*T. cruzi* clone R4J satellite sequence	2.00E−76	99.39
19	OM730059.1	*T. cruzi* clone R9J satellite sequence	2.00E−75	99.39
20	OM730061.1	*T. cruzi* clone R10I satellite sequence	2.00E−76	99.39
21	OM730055.1	*T. cruzi* clone R8I satellite sequence	2.00E−75	99.39
22	OM730038.1	*T. cruzi* clone M5 satellite sequence	1.00E−77	100.00
23	LT220332.1	*T. cruzi* microsatellite DNA locus cl16.1, clone Las Palomas 163	1.00E−72	98.17
24	OM730070.1	*T. cruzi* clone R26E satellite sequence	3.00E−74	98.78
26	LT220336.1	*T. cruzi* microsatellite DNA locus cl20.1, clone Las Palomas 163	6.00E−76	99.39

Sequences obtained from PCR amplicons were aligned with GenBank reference sequences to determine organism identity. E-values and percent identity values from BLASTn analyses are shown for each positive triatomine sample.

Although the sequence similarities suggest a possible affiliation with TcI, compatible with the findings by Rodriguez [20], it is essential to note that the nuclear satellite DNA (SatDNA) target used in this study is not a validated marker for DTU classification. Therefore, genotyping assumptions cannot be definitively made based on SatDNA alone. Additional genotyping using DTU-informative markers would be required to accurately characterize the *T. cruzi* strains circulating in triatomine vectors in this region.

### Geographic distribution of collected triatomines

**Figure 2.**
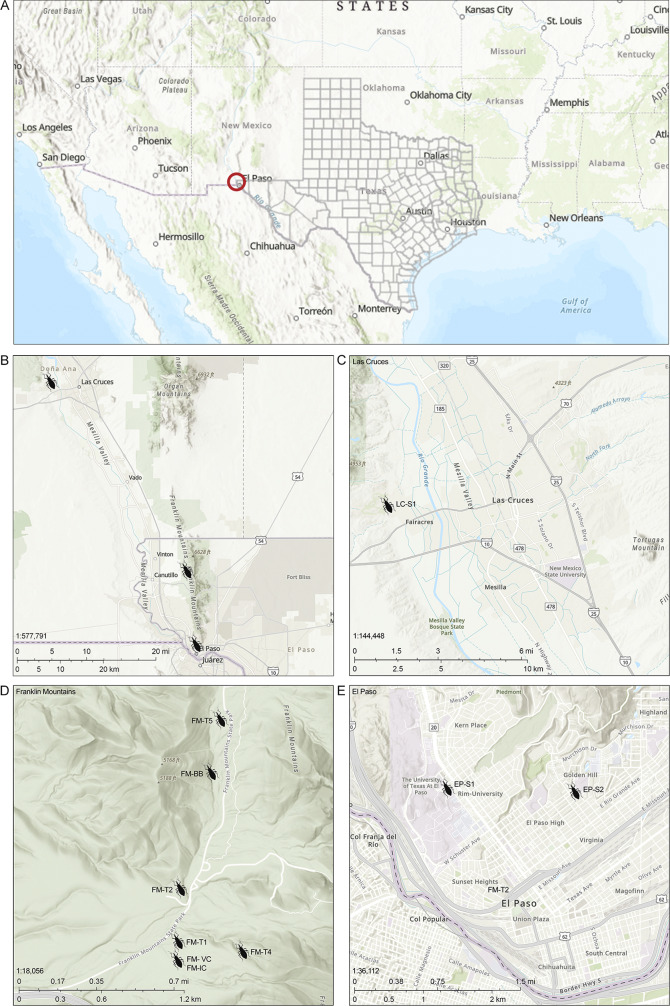
Geographic distribution of *T. rubida* collection sites in El Paso County, Texas, and Las Cruces in southern New Mexico. (A) Overview map showing the broader geographic context of El Paso, TX, near the U.S.–Mexico border, with a red circle indicating the focal region for triatomine collection. (B) Overview map showing the location of sampling sites across El Paso, TX, and Las Cruces, NM. (C) Close-up of Las Cruces, New Mexico, showing the location of Las Cruces Site 1 (LC-S1), where an adult *T. rubida* was collected in a semi-rural setting. (D) Detailed view of Franklin Mountains State Park in El Paso, Texas, showing sylvatic collection sites, including FM-T1 through FM-T5 and the Bird Blind site (FM-BB), where triatomines were retrieved from rocky and shrubby habitats. (E) Urban collection sites in El Paso, including EP-S1 and EP-S2, are located in residential areas where specimens were found under outdoor structures and in shaded microhabitats. Maps were generated using publicly available base layers and coordinates recorded during field collection. The spatial clustering of infected triatomines highlights both sylvatic and urban interfaces where potential vector-host interactions may occur.

*Triatoma rubida* specimens were collected from nine distinct sites across El Paso County, Texas, and southern New Mexico (Figure 2B). One site in southern New Mexico, designated Las Cruces Site 1 (LC-S1), yielded an infected triatomine and is located within a semi-rural environment near the city of Las Cruces (Figure 2C). Nevertheless, the majority of specimens were collected within Franklin Mountains State Park (Figure 2D), including locations such as the Bird Blind (FM-BB), Interpretive Center (FM-IC), Visitor Center (FM-VC), and multiple transect sites (FM-T1 through FM-T5). These sites represent sylvatic and peri-urban environments within a protected natural area frequented by both wildlife and human visitors. Additional specimens were obtained from two urban collection sites in El Paso (EP-S1 and EP-S2; Figure 2E), situated in proximity to residential areas and major urban infrastructure.

This spatial distribution highlights the presence of *T. cruzi*-positive triatomines in both sylvatic and urbanized areas, emphasizing the potential for human-vector contact not only in natural parks but also in residential zones. The clustering of positive specimens within Franklin Mountains suggests sustained local transmission, while the detection of positive bugs in Las Cruces and urban El Paso indicates wider geographic spread and potential cross-border public health relevance. The detection of *T. rubida* in these densely populated areas highlights a concerning migration of infected vectors from natural environments into urban settings, where the risk of contact with humans and domestic animals is significantly increased. This shift emphasizes the importance of monitoring vector presence at the urban–sylvatic interface, particularly in rapidly expanding metropolitan regions like El Paso.

## Discussion

This study provides an updated assessment of *T. cruzi* infection in *T. rubida* collected from El Paso County, Texas, and southern New Mexico, with a focus on the increasing presence of infected vectors in urban areas. In the present study, a total of 26 adult triatomines were collected over a 10-month surveillance period spanning from April 2024 to March 2025. All specimens were identified as *T. rubida*, and no nymphs were detected. Triatomines were captured from both sylvatic and urban environments. These findings stand in contrast to a previous surveillance effort conducted by Rodriguez et al. [20] in El Paso County [20], which reported the collection of 95 triatomines from 27 sites between 2016 and 2018. In that study, three triatomine species were documented: *T. rubida* (majority), *T. protracta*, and *T. gerstaeckeri.* Furthermore, 39% of the specimens collected were nymphs, whereas 61% were adults, suggesting active breeding sites and a broader representation of life stages. Importantly, approximately 30.5% of the collected triatomines were infected with *T. cruzi*, indicating the presence of both sylvatic and peridomestic transmission cycles in the region. In the present study, among the 26 triatomines tested, 84.6% were positive for *T. cruzi* by qPCR. This prevalence is notably higher than the 63.3% infection rate reported in our previous regional study [20], representing an increase of 21.3%. This change may reflect improved molecular detection, ecological or climatic shifts, or a true intensification of *T. cruzi* transmission.

The detection of infected vectors at both sylvatic sites, such as Franklin Mountains State Park, and urban or peri-urban locations in El Paso and Las Cruces stresses the adaptability of *T. cruzi* transmission cycles. This finding reinforces concerns about increased opportunities for human-vector and domestic animal-vector interactions in residential areas, with parallel trends reported across the southern United States [9, 11, 12, 28–30]. Additionally, these findings are especially relevant when considered in the broader biogeographical context. Mexico is recognized as one of the most triatomine-diverse countries, with more than 30 confirmed species and new ones being described [17]. This diversity, coupled with close ecological and human mobility links across the border, underscores the need for continuous vector surveillance and educational outreach in U.S. states bordering Mexico.

Sequencing of satellite DNA from positive triatomine samples confirmed *T. cruzi* identity and showed strong alignment to reference clones associated with the TcI lineage, the predominant discrete typing unit (DTU) in North and Central America [31, 32]. However, definitive DTU genotyping was not feasible due to low parasite DNA concentration; the use of SatDNA is not validated for genotyping. While the sequences suggest a likely affiliation with TcI, confirmation requires DTU-specific markers. These findings are consistent with previous studies in the region, which had shown the predominance of TcI in sylvatic, domestic, and peridomestic cycles [20]. To address this setback, ongoing efforts in our group aim to genotype *T. cruzi* isolates from collected vectors using multilocus sequence typing (MLST) and other nuclear markers to better characterize the discrete typing units (DTUs) circulating in the region [33]. These approaches will provide a more comprehensive understanding of parasite diversity and its potential implications for transmission dynamics and pathogenicity. In parallel, serological surveillance for *T. cruzi* infection has also been conducted in human populations residing in the El Paso–Las Cruces area. These data are currently being analysed and will be reported in a forthcoming publication. Together, these complementary efforts aim to enhance our understanding of both vector and host components of Chagas disease transmission at the U.S.–Mexico border.

The migration of infected *T. rubida* into urban El Paso neighbourhoods represents a significant public health concern, particularly given the limited awareness and diagnostic infrastructure for Chagas disease in the United States [7, 34]. Despite growing evidence of autochthonous transmission, Chagas disease remains underdiagnosed and underreported due to the lack of mandatory reporting and low clinical suspicion among healthcare providers [8].

In conclusion, this study highlights the continued presence and suggestive intensification of *T. cruzi* transmission among triatomine vectors in the El Paso–Las Cruces region that deserves to be further explored. The findings emphasize the importance of sustained vector surveillance, molecular characterization of circulating *T. cruzi* strains, and improved public health education to mitigate the risk of human infection [35]. Targeted outreach and veterinary screening programmes in high-risk communities should be considered essential components of local disease prevention strategies.

## Supporting information

10.1017/S095026882510071X.sm001 Farani et al. supplementary material Farani et al. supplementary material

## Data Availability

All data and materials supporting the findings of this study are included within the article and its supplementary materials.

## Supplementary material

The supplementary material for this article can be found at http://doi.org/10.1017/S095026882510071X.
